# Dissecting the Transcriptomes of Multiple Metronidazole-Resistant and Sensitive *Trichomonas vaginalis* Strains Identified Distinct Genes and Pathways Associated with Drug Resistance and Cell Death

**DOI:** 10.3390/biomedicines9121817

**Published:** 2021-12-02

**Authors:** Po-Jung Huang, Ching-Yun Huang, Yu-Xuan Li, Yi-Chung Liu, Lichieh-Julie Chu, Yuan-Ming Yeh, Wei-Hung Cheng, Ruei-Ming Chen, Chi-Ching Lee, Lih-Chyang Chen, Hsin-Chung Lin, Shu-Fang Chiu, Wei-Ning Lin, Ping-Chiang Lyu, Petrus Tang, Kuo-Yang Huang

**Affiliations:** 1 Department of Biomedical Sciences, Chang Gung University, Taoyuan City 333, Taiwan; pjhuang@mail.cgu.edu.tw; 2 Genomic Medicine Core Laboratory, Chang Gung Memorial Hospital, Linkou, Taoyuan City 333, Taiwan; ymyeh@mail.cgu.edu.tw (Y.-M.Y.); chichinglee@cgu.edu.tw (C.-C.L.); 3 Graduate Institute of Medical Sciences, National Defense Medical Center, Taipei City 114, Taiwan; Kelly12210422@gmail.com (C.-Y.H.); A3183@tpech.gov.tw (S.-F.C.); 4 Host-Parasite Interactions Laboratory, National Defense Medical Center, Taipei City 114, Taiwan; 5 Graduate Institute of Biomedical Sciences, College of Medicine, Chang Gung University, Taoyuan City 333, Taiwan; holmes0527@gmail.com (Y.-X.L.); julie.chu@mail.cgu.edu.tw (L.-J.C.); petang@mail.cgu.edu.tw (P.T.); 6 Institute of Bioinformatics and Structural Biology, Department of Life Science, National Tsing Hua University, Hsinchu 300, Taiwan; jong212@gmail.com (Y.-C.L.); pclyu@mx.nthu.edu.tw (P.-C.L.); 7 Molecular Medicine Research Center, Chang Gung University, Taoyuan City 333, Taiwan; 8 Liver Research Center, Chang Gung Memorial Hospital, Linkou, Taoyuan City 333, Taiwan; 9 Department of Medical Laboratory Science, College of Medicine, I-Shou University, Kaohsiung City 824, Taiwan; whcheng@isu.edu.tw; 10 Division of Clinical Pathology, Department of Pathology, Tri-Service General Hospital, National Defense Medical Center, Taipei City 114, Taiwan; j1885s@msn.com (R.-M.C.); hsinchunglin@gmail.com (H.-C.L.); 11 Department of Computer Science and Information Engineering, Chang Gung University, Taoyuan City 333, Taiwan; 12 Department of Medicine, Mackay Medical College, New Taipei City 252, Taiwan; lihchyang@mmc.edu.tw; 13 Graduate Institute of Pathology and Parasitology, National Defense Medical Center, Taipei City 114, Taiwan; 14 Department of Inspection, Taipei City Hospital, Renai Branch, Taipei City 114, Taiwan; 15 Graduate Institute of Biomedical and Pharmaceutical Science, Fu Jen Catholic University, New Taipei City 242, Taiwan; 081551@mail.fju.edu.tw

**Keywords:** trichomoniasis, drug resistance, ABC transporter, ERAD

## Abstract

*Trichomonas vaginalis* is the causative agent of trichomoniasis, the most prevalent non-viral sexually transmitted infection worldwide. Metronidazole (MTZ) is the mainstay of anti-trichomonal chemotherapy; however, drug resistance has become an increasingly worrying issue. Additionally, the molecular events of MTZ-induced cell death in *T. vaginalis* remain elusive. To gain insight into the differential expression of genes related to MTZ resistance and cell death, we conducted RNA-sequencing of three paired MTZ-resistant (MTZ-R) and MTZ-sensitive (MTZ-S) *T. vaginalis* strains treated with or without MTZ. Comparative transcriptomes analysis identified that several putative drug-resistant genes were exclusively upregulated in different MTZ-R strains, such as ATP-binding cassette (ABC) transporters and multidrug resistance pumps. Additionally, several shared upregulated genes among all the MTZ-R transcriptomes were not previously identified in *T. vaginalis*, such as 5′-nucleotidase surE and Na^+^-driven multidrug efflux pump, which are a potential stress response protein and a multidrug and toxic compound extrusion (MATE)-like protein, respectively. Functional enrichment analysis revealed that purine and pyrimidine metabolisms were suppressed in MTZ-S parasites upon drug treatment, whereas the endoplasmic reticulum-associated degradation (ERAD) pathway, proteasome, and ubiquitin-mediated proteolysis were strikingly activated, highlighting the novel pathways responsible for drug-induced stress. Our work presents the most detailed analysis of the transcriptional changes and the regulatory networks associated with MTZ resistance and MTZ-induced signaling, providing insights into MTZ resistance and cell death mechanisms in trichomonads.

## 1. Introduction

The protozoan parasite *Trichomonas vaginalis* is the causative agent of human trichomoniasis and annually infects approximately 276 million people worldwide [1]. *T. vaginalis* colonizes the urogenital tract of humans and mainly leads to vaginal discharge, pruritus, odor, and irritation in women [2]. Importantly, the complications of trichomoniasis are accompanied by severe health consequences, such as cervical cancer [3], adverse pregnancy outcomes [4], infertility [5], and human immunodeficiency virus (HIV) transmission [6]. Although men are often asymptomatic carriers of *T. vaginalis*, dysuria, discharge, and increased risk of prostate cancer have been reported [7]. The nitroimidazole drug family, represented by MTZ and tinidazole (TNZ), is the only class of drugs currently approved by the Food and Drug Administration for the treatment of trichomoniasis. However, the reliance on a single therapeutic class is problematic, since MTZ-R isolates are on the rise [8,9]. A previous study stated that 4.3% of *T. vaginalis* isolates from multiple geographic sites in the United States display MTZ resistance [10]. Although higher oral MTZ doses can sometimes cure refractory trichomoniasis, the side effects during treatment appeared to be relatively common [11]. It is necessary to develop alternative therapeutic agents against MTZ-R *T. vaginalis*.

MTZ is a prodrug and must be reduced to its active nitro group to form radical anions, which are toxic to parasites. Aerobic and anaerobic resistance to MTZ has been demonstrated in *T. vaginalis*. Aerobic resistance, also called clinical resistance, is achieved by re-oxidizing nitroradical anions in the presence of oxygen to decrease the activity of MTZ, which does not downregulate the transcription of hydrogenosomal pyruvate: ferredoxin oxidoreductase (PFOR) and ferredoxin (Fd) [12,13]. In contrast, anaerobic resistance is characterized by the absence of PFOR and Fd [14,15]. Flavodoxin reductase (FR) is an oxygen scavenging enzyme, and downregulation of FR1 has been considered to mediate both anaerobic and aerobic resistance [16,17]. Iron supplementation has also been shown to play a role in MTZ susceptibility in *T. vaginalis* and the expression levels of iron-regulated genes, such as zinc regulated transporter, iron regulated transporter-like gene (*TvZIP4*), Fe–S cluster assembly gene (*TvIscA*), and Fe-containing superoxide dismutase gene (*TvFeSOD*), change significantly in MTZ-R parasites [18]. Another study has identified and characterized single nucleotide polymorphisms (SNPs) in two nitroreductase genes (*ntr4Tv* and *ntr6Tv*) associated with MTZ resistance [19]. While previous studies on MTZ resistance mechanisms in *T. vaginalis* mainly focus on the genes involved in oxygen stress response and hydrogenosomal metabolism, identifying additional candidate genes or pathways in various MTZ-R strains may extend our knowledge of drug resistance in trichomonads.

High-throughput multi-omics approaches provide an excellent opportunity to dissect the heavily duplicated genome of *T. vaginalis* that contains about ~60,000 predicted protein-coding genes [20], which are helpful to uncover novel genes or proteins modulating drug resistance. For example, genome sequencing of multiple *T. vaginalis* clinical isolates identified 72 SNPs potentially associated with drug resistance [21]. Additionally, transcriptomic profiles of several MTZ-R strains showed common changes in genes involved in drug activation, accumulation, and detoxification [21]. We previously identified that amino-acid-related metabolisms are the most upregulated pathways in MTZ-R parasites upon MTZ treatment at the proteome level, whereas oxidative phosphorylation is chiefly downregulated [22]. Hence, a large-scale genetic comparison of MTZ-S and MTZ-R strains treated with or without MTZ using comprehensive omics approaches will identify novel mechanisms governing drug resistance in *T. vaginalis*.

While increased MTZ resistance has emerged as a highly problematic public health issue and the progress in studying drug resistance mechanisms in *T. vaginalis* is slow, we aim to identify novel molecular mechanisms of MTZ resistance by conducting comparative transcriptomic analysis of multiple MTZ-R and MTZ-S strains using RNA sequencing (RNA-seq). Additionally, altered transcriptomes of MTZ-S parasites treated with MTZ represent responses of parasites to drug-induced stress or cell death stimuli. We have identified previously uncharacterized genes and enriched biological pathways in parasites associated with MTZ resistance and stress response, significantly advancing our understanding of the transcriptional regulatory networks concerning drug resistance and cell death in trichomonads.

## 2. Materials and Methods

### 2.1. T. vaginalis Strains and Cell Culture

The *T. vaginalis* MTZ-S strains (ATCC 30001, ATCC 30236, and ATCC50148) and MTZ-R strains (ATCC 50142, ATCC 50143, and ATCC 30238) were maintained in YIS medium, pH 5.8, containing 10% heat-inactivated horse serum and 1% glucose at 37 °C. The trypan blue exclusion assay monitored the growth of the parasites.

### 2.2. MTZ Susceptibility Assay of Various T. vaginalis Strains

To confirm the MTZ susceptibility of all tested strains, the cell density of MTZ-S and MTZ-R isolates was monitored every 4 h by the trypan blue exclusion assay after treatment with different concentrations of MTZ (5, 10, 20 μM) (Sigma-Aldrich, Saint Louis, MO, USA) compared with that of the sterile distilled water (SDW)-treated control. The initial concentration of MTZ-S and MTZ-R strains was 2 × 10^5^ cells/mL. The concentration and incubation time of MTZ (20 μM for 8 h) for transcriptomic analysis was determined as previously described [22].

### 2.3. RNA Extraction and Quality Assessment

Total RNA was extracted from MTZ-S and MTZ-R parasites treated with MTZ (20 μM) or SDW for 8 h using the TRI Reagent^®^ (Molecular Research Center). The quality and quantity of the RNA samples were verified using both a Nanodrop spectrophotometer and a Qubit fluorometer. The RNA integrity number (RIN) score was calculated for each sample with a Caliper LabChip analyzer. Samples with RIN scores greater than 7 were considered suitable for subsequent analysis.

### 2.4. RNA-Seq and Bioinformatics Analysis

A total of 36 RNA samples isolated from six MTZ-S and MTZ-R strains treated with or without MTZ (20 μM) were analyzed by RNA-seq. The cDNA libraries were generated using Illumina TruSeq RNA Sample Preparation kits v2 and sequenced by the NextSeq 500 system. Cutadapt, FastQC, and MultiQC [23,24] were applied to the resulting FASTQ files to improve the quality of sequences by trimming adapters and filtering low-quality bases (Q < 20), create a report of sequencing quality, and aggregate reports from each quality control step, respectively. STAR was utilized to create the genome index and map reads to the reference genome of *T. vaginalis*. FeatureCounts [25] was used to quantify the number of reads per annotated gene across different strains and conditions. Identification of differentially expressed genes (DEGs) was performed using the DESeq2 package [26], and the relative abundances of transcripts were shown as normalized counts. In DESeq2, the *p*-values attained by the Wald test were corrected for multiple testing using the Benjamini and Hochberg method. DEGs with false discovery rate (FDR) < 0.05 and |log2 fold change in normalized counts| ≥1 were considered significant. R packages such as ComplexHeatmap [27] and clusterProfiler [28] were adopted to generate the heatmap of the normalized counts of genes in individual samples and perform the functional enrichment analysis of the DEGs.

### 2.5. Homology Model

The protein sequence of MdaB (TVAG_091830) was retrieved from the National Center for Biotechnology Information (NCBI) protein sequence database (http://www.ncbi.nlm.nih.gov/protein, assessed on 3 October 2021) and subsequently searched against the sequence database of RCSB Protein Data Bank (PDB) [29] by PSI-BLAST [30], resulting in a template structure with 46% sequence identity and covering 93% of the query sequence (PDB ID: 3RPE). We utilized the SWISS-MODEL server (http://swissmodel.expasy.org/, assessed on 3 October 2021) to construct the 3D structure of the target protein. The quality of the protein model was evaluated by Ramachandran plot and root-mean-square deviation (RMSD) analyses using PROCHECK [31] and the RMSD calculator tool of Swiss-PDB viewer [32], respectively. Furthermore, the ERRAT tool [33] and ProSA [34] were applied to examine the quality of the modeled structure based on a series of qualitative assessment methods.

### 2.6. Protein-Ligand Docking Analysis

PyRx version 0.97 and AutoDock Vina were used for molecular docking [35]. The three-dimensional (3D) structure of MTZ was retrieved from NCBI (PubChem CID: 4173). The search space parameters were adjusted to a grid box of 64.57 Å × 12.24 Å × 7.57 Å to cover the whole active site of the protein. According to their binding affinity (kcal/mol), nine docking results were selected as the best models for protein-ligand complexes. The PyMOL Molecular Graphics System (Ver. 2.2 Schrödinger, Portland, OR, USA) was used to display the 3D structures of the protein-ligand complexes.

## 3. Results

### 3.1. MTZ Susceptibility of Six MTZ-S and MTZ-R T. vaginalis Strains

**Figure 1:**
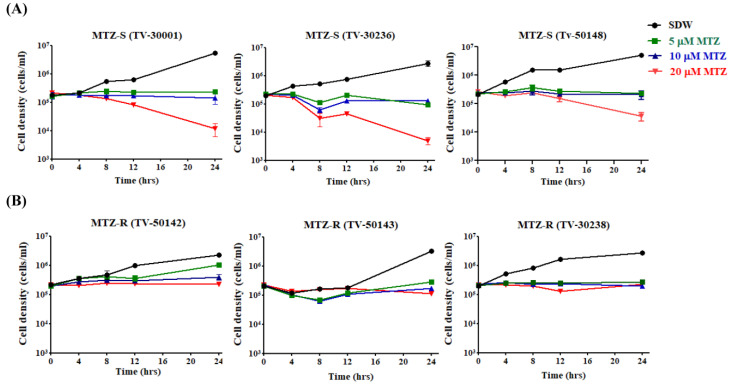
Validation of MTZ susceptibility of six MTZ-S and MTZ-R *T. vaginalis* isolates. The effect of MTZ on the growth of three MTZ-S (**A**) (Tv-30001, Tv-30236, and Tv50148) and three MTZ-R (**B**) (Tv-50142, Tv-50143, and Tv-30238) isolates was monitored. The initial concentration of MTZ-S and MTZ-R strains was 2 × 10^5^ cells/mL. MTZ-S and MTZ-R trophozoites were treated with different concentrations of MTZ (5, 10, or 20 μM) compared with the SDW-treated control. The cell density was monitored by the trypan blue exclusion assay. The growth curves were presented as mean ± SD of three independent experiments.

We validated the MTZ susceptibility of three MTZ-S (Tv-30001, Tv-30236, and Tv50148) and three MTZ-R strains (Tv-50142, Tv-50143, and Tv-30238) by treatment with different concentrations of MTZ (5, 10, and 20 μM) compared with the SDW-treated control. The cell density of all MTZ-S isolates treated with 20 μM of MTZ was significantly reduced after 24 h (Figure 1A), whereas the MTZ-R isolates maintained the viability under the same treatment (Figure 1B). Since 8 h of 20 μM MTZ treatment was able to differentiate the growth patterns between the MTZ-S and MTZ-R strains as previously described [22], we used this condition for subsequent comparative transcriptome analysis.

### 3.2. RNA-Seq of Six MTZ-S and MTZ-R T. vaginalis Strains in the Presence or Absence of MTZ

**Figure 2:**
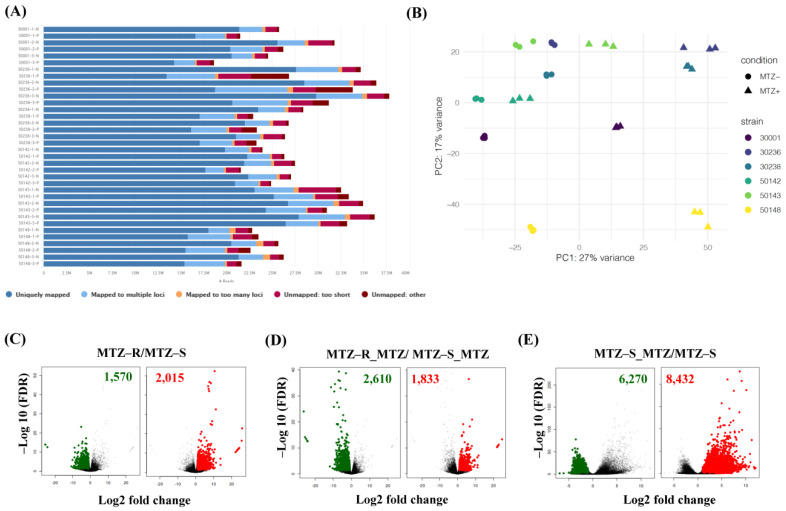
A global view of DEGs in the MTZ-R and MTZ-S transcriptomes treated with or without MTZ. (**A**) The reads generated from the RNA-seq data of six *T. vaginalis* isolates in the presence or absence of MTZ were mapped to the *T. vaginalis* reference genome, and the percentage of mapped reads was shown. (**B**) PCA of six isolates in the presence (MTZ+) or absence (MTZ-) of MTZ treatment. Biological triplicates of each sample were presented as different colors. Volcano plots showed the significantly differential gene expression (FDR < 0.05, log2 |fold change| = 1) in the MTZ-R transcriptomes compared with those of MTZ-S in the absence of MTZ (MTZ-R/MTZ-S) (**C**) or upon MTZ treatment (MTZ-R_MTZ/MTZ-S_MTZ) (**D**). (**E**) DEGs in the MTZ-S transcriptomes treated with MTZ was compared with those of MTZ-S (MTZ-S_MTZ/MTZ-S). The green and red dots represent the downregulated or upregulated genes in each group, respectively.

To assess the transcriptional changes associated with MTZ resistance, we conducted RNA-seq analysis to compare the transcriptomes of three MTZ-R and three MTZ-S isolates treated with (MTZ-R_MTZ vs. MTZ-S_MTZ) or without MTZ (MTZ-R vs. MTZ-S). To examine the changes in gene expression potentially associated with MTZ-induced stress response or cell death, the transcriptomes of MTZ-S isolates treated with MTZ were compared with those of the identical MTZ-S isolates without drug treatment (MTZ-S_MTZ vs. MTZ-S). The mRNA populations derived from biological triplicates of each high-quality RNA sample (RIN > 7.0) were sequenced and mapped to the *T. vaginalis* G3 genome. The average percentage of uniquely mapped reads for the 36 RNA samples was 76.41%, generating over 760 million uniquely mapped reads (Figure 2A). The general expression profile of each isolate was determined by principle component analysis (PCA), revealing good separation among different MTZ-S and MTZ-R isolates in the presence or absence of MTZ (Figure 2B). We globally analyzed the DEGs between the MTZ-S and MTZ-R transcriptomes in the presence or absence of MTZ using DESeq2 (FDR < 0.05, |log2 fold change| ≥ 1). Cluster analysis revealed 3585 DEGs in the MTZ-R group compared with the MTZ-S group, with 2015 upregulated and 1570 downregulated genes (Figure 2C and Supplementary Table S1). Additionally, there were 4443 DEGs in the MTZ-R transcriptomes treated with MTZ when compared with the MTZ-S group under the same treatment, with 1833 upregulated and 2610 downregulated genes (Figure 2D and Supplementary Table S2). This suggests that more genes are downregulated in MTZ-R parasites in response to MTZ treatment. Moreover, the MTZ-S isolates treated with MTZ expressed 14,072 DEGs relative to the untreated MTZ-S group, with 8432 upregulated and 6270 downregulated genes (Figure 2E and Supplementary Table S3), suggesting that a higher number of genes are upregulated in MTZ-S parasites in response to drug-induced stimuli.

### 3.3. Differential Expression of the Previously Identified Genes Involved in Drug Resistance in the MTZ-R Transcriptomes Compared with Those of MTZ-S

#### 3.3.1. Hydrogenosomal Metabolism

**Figure 3:**
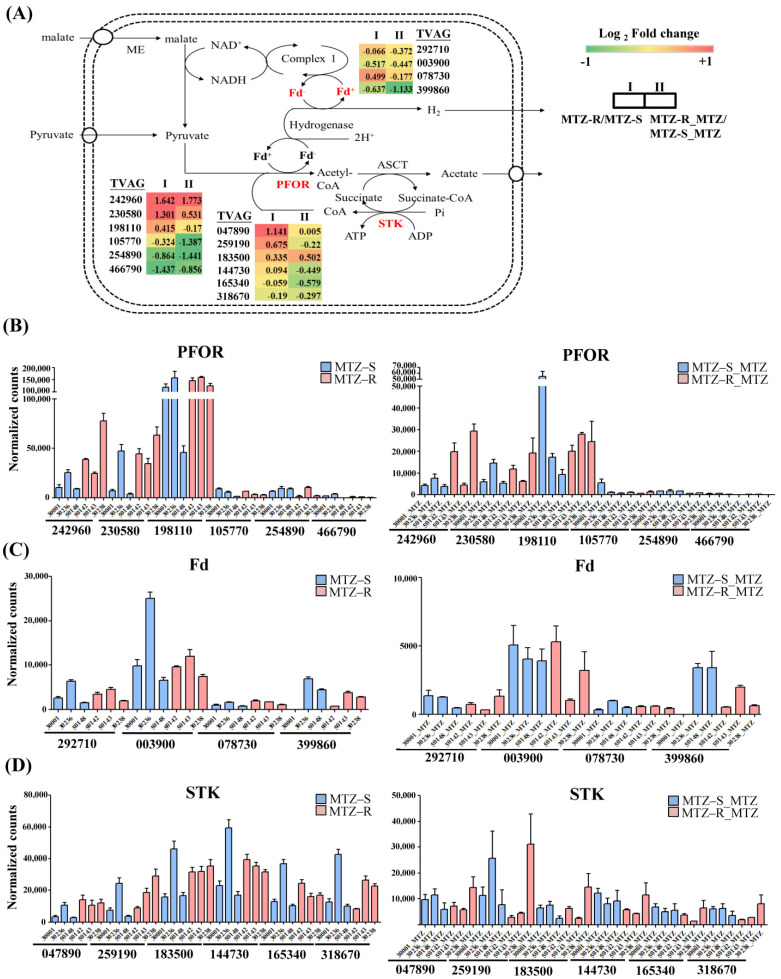
Differential expression of the hydrogenosomal genes in the MTZ-R *T. vaginalis* transcriptomes treated with or without MTZ. (**A**) Differential gene expression of PFOR, Fd, and STK at the isoform levels between the MTZ-R and MTZ-S transcriptomes treated with (MTZ-R_MTZ/MTZ-S_MTZ; II) or without MTZ (MTZ-R/MTZ-S; I) were presented as log2 fold change. Genes shown in red and green color indicate upregulation and downregulation, respectively. Gene expression levels of different (**B**) PFOR, (**C**) Fd, and (**D**) STK isoforms in the MTZ-R and MTZ-S transcriptomes treated with or without MTZ were presented as mean ± SD of normalized counts values obtained from three independent RNA-seq experiments.

Laboratory-generated MTZ resistance has been linked with the downregulation of specific hydrogenosomal enzymes, such as PFOR and Fd [12,13]. Hence, the differential expression of these genes involved in hydrogenosomal metabolism [20] was analyzed in the MTZ-R transcriptomes compared with those of MTZ-S in the presence or absence of MTZ (Supplementary Tables S4 and S5). There were three hydrogenosomal PFOR genes (TVAG_242960, TVAG_230580, and TVAG_198110) predominantly expressed in all MTZ-S and MTZ-R strains in the presence or absence of MTZ (Figure 3A,B). For these highly expressed PFOR, two of three were upregulated in the MTZ-R transcriptomes compared with those of MTZ-S treated with or without MTZ. Conversely, all the lower expressed PFOR genes (TVAG_105770, TVAG_254890, and TVAG_466790) were downregulated in the MTZ-R transcriptomes. Additionally, three and four Fd isoforms were downregulated in the MTZ-R transcriptomes in the absence and presence of MTZ, respectively (Figure 3C). We previously identified that succinate thiokinase (STK) proteins were downregulated in MTZ-R parasites (Tv-50143) treated with MTZ compared with that of MTZ-S parasites (Tv-30236) at the proteome level [22]. We herein found that all STK genes exhibited higher expression in the MTZ-S Tv-30236 strain than the rest of MTZ-S strains and MTZ-R parasites (Tv-50143) (Figure 3D). Upon MTZ treatment, four of six STK genes (TVAG_259190, TVAG_144730, TVAG_165340, and TVAG_318670) were downregulated in the MTZ-R transcriptomes. Together, the overall expression of these hydrogenosomal genes was suppressed in MTZ-S or MTZ-R parasites in response to MTZ treatment. Except that all Fd isoforms were downregulated in the MTZ-R transcriptomes upon MTZ treatment, several PFOR and Fd genes were downregulated in different MTZ-R strains, suggesting that downregulation of these hydrogenosomal genes may not be a consistent pattern in all MTZ-R strains.

#### 3.3.2. Oxygen Stress Response

Oxygen stress response genes, such as flavin reductase (FR), play crucial roles in MTZ resistance [16,17]. There are eight FR isoforms in the *T. vaginalis* genome [20], and downregulation of FR1 (TVAG_517010) has been proven to regulate MTZ resistance [16]. We found that FR1, FR2 (TVAG_311580), FR6 (TVAG_009980), and FR7 (TVAG_127310) were highly expressed, whereas FR5-2 (TVAG_144100) was nearly not expressed (normalized counts <10) in the MTZ-S and MTZ-R transcriptomes treated with or without MTZ (Supplementary Figure S1). Interestingly, it was evident that the expression levels of FR1 were upregulated in all MTZ-S strains and further enhanced upon MTZ treatment, thereby resulting in more downregulation in the MTZ-R strains. Hence, our data were consistent with the previous finding [17], indicating that FR1 was downregulated in MTZ-R parasites treated with or without MTZ. We also found that the expression levels of FR2 were mainly upregulated in the MTZ-S Tv-30236 and MTZ-R Tv-30238 strains, implying its unique function other than regulating drug resistance.

#### 3.3.3. Iron-Regulated Genes

Iron supplementation has been shown to link to an increase in the susceptibility of *T. vaginalis* to MTZ [18], suggesting that parasites may develop potential MTZ resistance mechanisms under iron-deficient (ID) environments. We next investigated whether iron-regulated genes [36] were differentially expressed in the MTZ-R transcriptomes in the presence or absence of MTZ. A previous study has identified 115 and 75 upregulated genes under iron-rich (IR) and ID conditions, respectively [36]. For the iron-regulated genes expressed in high and low iron environments, 15% and 23% of these genes were also upregulated (log2 fold change > 1) in the MTZ-R transcriptomes compared with those of MTZ-S (Supplementary Table S6). Conversely, 1% and 5% of the iron-regulated genes were downregulated (log2 fold change < −1) in the MTZ-R transcriptomes. Notably, we found that a lower proportion of the iron-regulated genes (10% and 11% for IR and ID conditions, respectively) were upregulated in the MTZ-R transcriptomes exposed to MTZ, whereas a higher proportion of these genes (6% and 11% for IR and ID conditions, respectively) were downregulated in this scenario (Supplementary Table S7), indicating that more iron-regulated genes were downregulated in MTZ-R parasites in response to drug treatment. Specifically, a Na^+^-driven multidrug efflux pump (TVAG_064460) was upregulated in the MTZ-R transcriptomes treated with or without MTZ, implying its potential role in drug resistance in MTZ-R parasites under IR conditions. Intriguingly, we noted that several ID-enriched genes potentially associated with drug resistance were also upregulated in the MTZ-R transcriptomes (Supplementary Table S6), including multidrug resistance (MDR) protein 1, 2 (TVAG_542460), MDR pump (TVAG_304360), and Na^+^-driven multidrug efflux pump (TVAG_436530), one of which (TVAG_304360) remained upregulated in the MTZ-R transcriptomes treated with MTZ. These results indicated that different paralogues of Na^+^-driven multidrug efflux pump were specifically regulated by IR and ID environments, suggesting distinct drug-resistant mechanisms in MTZ-R parasites in response to iron availability changes. We also provided the potential target genes that may regulate MTZ resistance in *T. vaginalis* upon iron deficiency.

### 3.4. Identification of Shared Upregulated or Downregulated Genes in All MTZ-R Strains

**Figure 4:**
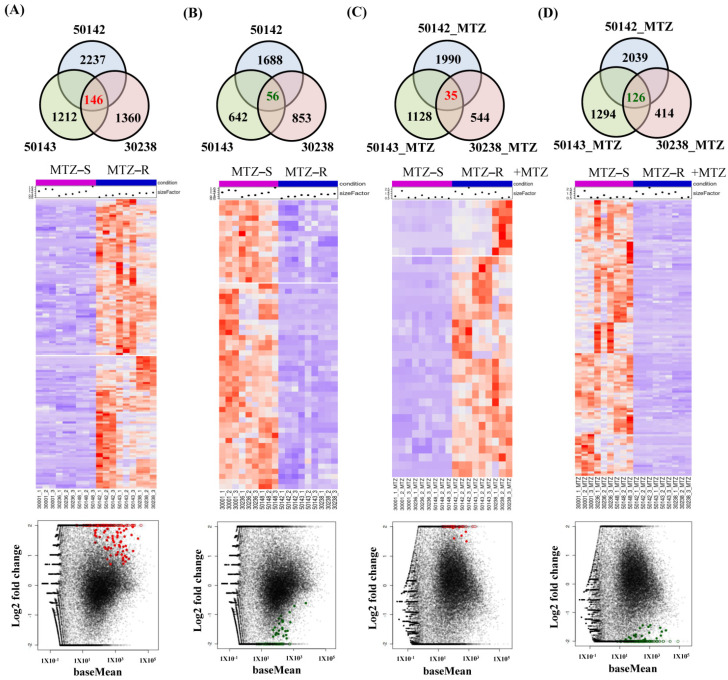
Identification of common DEGs in all MTZ-R *T. vaginalis* strains compared with the MTZ-S group treated with or without MTZ. Venn diagram showing the number of genes shared by three MTZ-R *T. vaginalis* strains (Tv_50142, Tv50143, and Tv30238) whose expression is upregulated (**A**) and downregulated (**B**) in comparison with three MTZ-S strains (Tv-30001, Tv-30236, and Tv50148). Upon MTZ treatment, the number of upregulated (**C**) and downregulated (**D**) genes among the identical MTZ-R strains in comparison with the MTZ-S strains were also analyzed. The gene expression patterns of DEGs in all MTZ-R strains compared with those of MTZ-S in the presence or absence of MTZ were analyzed by cluster analysis and shown as heatmaps. Genes shown in red and purple color indicate upregulation and downregulation, respectively. MA plots represent the expression levels (baseMean) and expression changes (log2 fold change in normalized counts values) in the corresponding DEGs.

**Figure 5:**
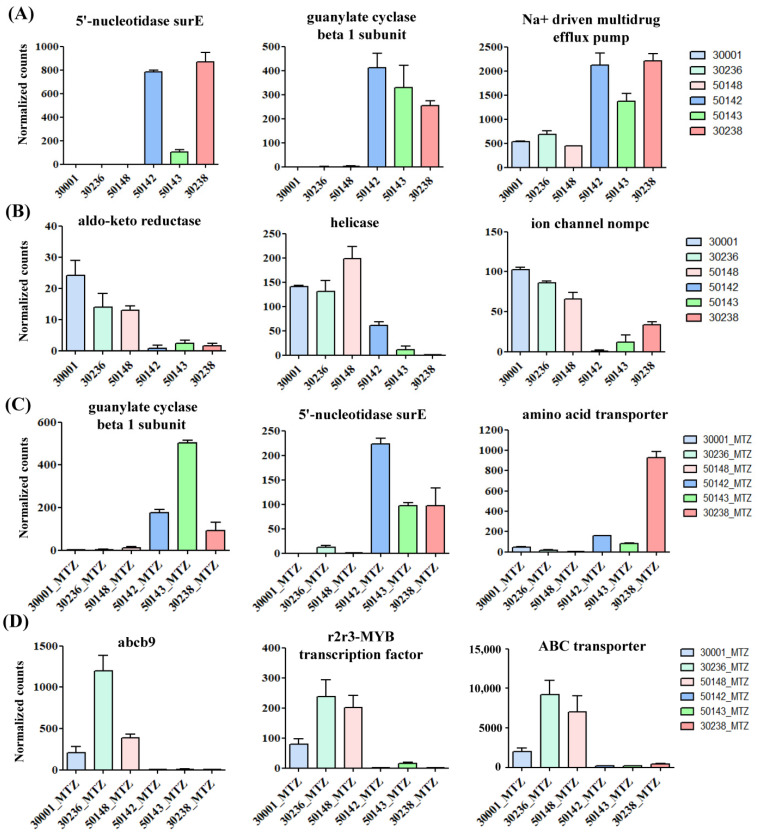
Commonly upregulated and downregulated genes in the MTZ-R *T. vaginalis* transcriptomes treated with or without MTZ. The selected genes were observed to be upregulated (**A**) and downregulated (**B**) in the MTZ-R transcriptomes (50142, 50143, and 30238) compared with those of MTZ-S (30001, 30236, and 50148). Upon MTZ treatment, the selected upregulated (**C**) and downregulated (**D**) genes in the same MTZ-R strains in comparison with the MTZ-S strains were also analyzed. Gene expression levels were presented as mean ± SD of normalized count values obtained from three independent RNA-seq experiments.

To identify the common upregulated or downregulated genes among all the MTZ-R strains, we compared each of the three MTZ-R strains with the MTZ-S group comprising three strains. All the MTZ-R strains shared 146 upregulated and 56 downregulated genes (Figure 4A,B and Supplementary Table S8) relative to the MTZ-S group. Additionally, all the MTZ-R strains shared 35 upregulated and 126 downregulated genes in response to MTZ treatment compared with the MTZ-S group (Figure 4C,D and Supplementary Table S9). The shared DEGs of all MTZ-R strains treated with or without MTZ were shown as heatmaps and MA plots to ensure their expression patterns (Figure 4A–D). We highlighted the shared DEGs with functional annotations in Table 1 and Table 2. We found that 5′-nucleotidase surE (TVAG_195930) was the most upregulated gene in the MTZ-R group (log2 fold change = 11.2) and remained significantly upregulated in response to MTZ treatment (log2 fold change = 5.1), whereas it was almost not expressed in all MTZ-S strains (Figure 5A,C). Additionally, there were eight genes encoding for leucine-rich repeat proteins of the BspA family that were upregulated in all MTZ-R strains when compared with the MTZ-S group, and one of them (TVAG_396470) remained upregulated in all MTZ-R strains in response to MTZ treatment (Table 1 and Table 2). Moreover, guanylate cyclase beta 1 subunit (TVAG_219680) was significantly upregulated in all MTZ-R strains treated with or without MTZ (log2 fold change = 5.8 and 8.5, respectively) (Figure 5A,C). The expression of amino acid transporter (TVAG_019720) was further induced in all MTZ-R strains treated with MTZ (Figure 5C). It is noteworthy that Na^+^-driven multidrug efflux pump (TVAG_483040) and its paralogue (TVAG_254920) were upregulated in all MTZ-R strains in the absence and presence of MTZ, respectively (Figure 5A, Table 1 and Table 2), suggesting their potential roles in MTZ resistance.

On the other hand, several shared downregulated genes in all MTZ-R strains treated with or without MTZ were also previously uncharacterized. For example, aldo-keto reductase (TVAG_048690 and TVAG_602270) and helicase (TVAG_524510) were significantly downregulated in all MTZ-R strains in the presence or absence of MTZ (Figure 5B, Table 1 and Table 2). Interestingly, the members of ABC transporters, such as abcb9 (TVAG_078520) and the ABC transporter (TVAG_431960) were strikingly downregulated in all MTZ-R strains upon MTZ treatment (Figure 5D and Table 2). It is noteworthy that the expression of abcb9 was exclusively induced in all MTZ-S strains and nearly not expressed (RPKM < 10) in all MTZ-R strains, suggesting its role in drug susceptibility. Other transporters, such as sugar transporter (TVAG_152410) and zinc–iron transporter (TVAG_415420), were also downregulated in all MTZ-R strains upon MTZ treatment (Table 2). The expression levels of several transcription factors were negatively regulated in the MTZ-R strains treated with MTZ, including r2r3-MYB transcription factor (TVAG_273350 and TVAG_318200) and MYB-1 (TVAG_328000) (Figure 5D and Table 2). PFOR (TVAG_254890) and alcohol dehydrogenase (TVAG_329660), whose expressions were previously identified to be downregulated in MTZ-R parasites [17] and proven to be associated with MTZ resistance [14], were also downregulated in all MTZ-R strains treated with MTZ (Table 2), supporting their roles in drug resistance. Thus, the roles of these common DEGs identified from multiple MTZ-R strains deserve further investigations and may develop shared MTZ resistance mechanisms in trichomonads.

### 3.5. Identification of Uniquely Upregulated Genes in Each MTZ-R and MTZ-S Strain

Isotype-specific MTZ resistance mechanism has been demonstrated in different MTZ-R *Giardia duodenalis* lines [37]. Based on the cluster analysis (FDR < 0.05, |log2 fold change| ≥1) of the MTZ-R and MTZ-S transcriptomes in the presence or absence of MTZ, we observed the distinct expression patterns in different MTZ-R and MTZ-S strains. We then analyzed the exclusively expressed genes in each MTZ-R strain to identify the potential strain-specific drug resistance mechanisms (Supplementary Table S10). We found that several genes were solely upregulated in the MTZ-R Tv-50142 strain, including ABC transporters (TVAG_162060 and TVAG_024550), MDR pump (TVAG_369180), MdaB (TVAG_091830), and leucine-rich repeat proteins, BspA family, whereas these genes were nearly not expressed (normalized counts <10) in the other MTZ-S or MTZ-R strains. In the MTZ-R Tv-50143 strain, ABC transporter (TVAG_478620), guanylate cyclase (TVAG_373630), and several leucine-rich repeat proteins, BspA family, were distinctly expressed. Moreover, inorganic phosphate transporter (TVAG_245560), ABC transporter (TVAG_241640), and sugar transporter (TVAG_145620) were specifically expressed in the MTZ-R Tv-30238 strain. Likewise, the same ABC transporter (TVAG_162060) and leucine-rich repeat proteins, BspA family, maintained their expression in the MTZ-R Tv-50142 strain in response to MTZ treatment (Supplementary Table S11). In the MTZ-R Tv-50143 strain treated with MTZ, the ABC transporter (TVAG_478620) remained distinctly expressed, and the expression levels of another two ABC transporters (TVAG_506660 and TVAG_006570) were further induced by MTZ. The MTZ-R Tv30238 strain treated with MTZ retained the expression of the same genes encoding for inorganic phosphate transporter and ABC transporter. These results suggest that specific genes uniquely expressed in each MTZ-R strain also maintained their expression in response to drug treatment, and several of them are different isoforms of ABC transporters. Conversely, we analyzed the particularly expressed genes in each MTZ-S strain (Supplementary Table S12). It is noteworthy that MdaB (TVAG_184640, TVAG_184660, and TVAG_011570) were only expressed in the MTZ-S Tv-50148 strain. The three isoforms of MdaB further enhanced their expressions in the MTZ-S Tv-50148 strain treated with MTZ, suggesting their potential roles in MTZ susceptibility (Supplementary Table S13). In summary, these results not only identified specific biomarkers expressed in each MTZ-S and MTZ-R strain but implied that various drug resistance mechanisms might exist in different MTZ-R strains.

### 3.6. Functional Enrichment Analysis of MTZ-S Parasites Treated with MTZ Identifies Novel Genes and Pathways Associated with Drug-Induced Stress Responses

**Figure 6:**
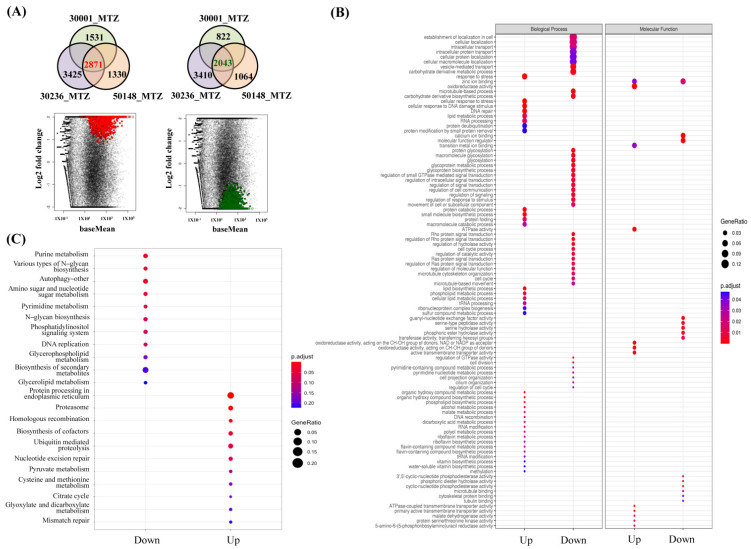
Functional enrichment analysis of DEGs in MTZ-S transcriptomes treated with MTZ compared with those of the untreated MTZ-S group. (**A**) Venn diagram showing the number of DEGs shared by three MTZ-S *T. vaginalis* strains treated with MTZ (Tv-30001_MTZ, Tv-30236_MTZ, and Tv50148_MTZ), whose expressions are upregulated (2871 genes) and downregulated (2043 genes) in comparison with the untreated MTZ-S parasites. MA plots represent the expression levels (baseMean) and expression changes (log2 fold change in normalized counts values) in the corresponding DEGs. (**B**) GO enrichment analysis of DEGs shared by three MTZ-S strains treated with MTZ compared with the untreated MTZ-S parasites. A significant upregulation and downregulation was observed in GO categories that include biological process and molecular function. (**C**) KEGG enrichment analysis of the DEGs shared by three MTZ-S strains treated with MTZ compared with the untreated MTZ-S parasites. A significant upregulation and downregulation were observed based on KEGG pathways. Dot size represents the ratio of genes in each category, and color indicates the adjusted *p*-value.

As MTZ-S strains are susceptible to MTZ, we could determine the significant differences in gene expression potentially involved in cell death in MTZ-S parasites treated with MTZ. All MTZ-S strains treated with MTZ shared 2871 upregulated genes and 2043 downregulated genes compared with the untreated MTZ-S group (Figure 6A and Supplementary Tables S14 and S15). It is worth mentioning that MdaB (TVAG_357140 and TVAG_357090) were the most upregulated genes in the MTZ-S strains treated with MTZ, especially in the Tv-50148 strain, and they were almost not expressed (normalized counts <10) in the strains without drug exposure. Additionally, over 60 tRNAs that bear different types of anticodons were distinctly upregulated in the MTZ-S strains treated with MTZ, especially in the Tv-30236 and Tv-50148 strains, suggesting that these genes have potential roles in MTZ-induced stress or cell death stimuli.

To investigate the enriched biological processes in MTZ-S parasites in response to MTZ treatment, we analyzed the common DEGs in the MTZ-S transcriptomes treated with MTZ using Gene Ontology (GO) and Kyoto Encyclopedia of Genes and Genomes (KEGG) enrichment analysis. GO terms showed that several biological processes related to stress responses were significantly enriched in MTZ-S parasites treated with MTZ, such as cellular response to stress, cellular response to DNA damage stimulus, and DNA repair (Figure 6B and Supplementary Table S16). Additionally, tRNA processing and tRNA modification were enriched, supporting that many tRNAs were upregulated in parasites in this scenario. Molecular function categories revealed that oxidoreductase activity, ATPase-coupled transmembrane transporter activity, and malate dehydrogenase were the most upregulated pathways in MTZ-treated parasites (Figure 6B). KEGG pathway mapping showed that protein processing in endoplasmic reticulum, proteasome, ubiquitin-mediated proteolysis, and mismatch repair were the most upregulated pathways, whereas those involved in purine and pyrimidine metabolism, DNA replication, and autophagy were downregulated (Figure 6C and Supplementary Table S17). The detailed expression patterns of individual genes participating in these enriched pathways were further analyzed (Supplementary Figures S2–S6). Intriguingly, we noted that genes involved in ERAD were strikingly upregulated in MTZ-S parasites treated with MTZ, which was concomitant with enhanced expression of genes encoding proteins for proteasome and ubiquitin-mediated proteolysis (Supplementary Figure S7). Collectively, these results demonstrate that MTZ treatment suppresses nucleotide metabolisms of parasites, which is accompanied by robust activation of multiple cytoprotective responses; however, the ultimate consequence of cell death cannot be altered.

### 3.7. Molecular Docking Studies for the Identification of Novel MTZ-Binding Proteins

Next, we attempted to identify the potential MTZ-binding protein targets from the DEGs mentioned above using the combination of protein modeling and molecular docking methods. Since three-dimensional protein structure is evolutionarily more conserved than primary protein sequence, the protein sequence encoded by a putative MdaB (TVAG_091830) displayed 46% sequence identity with the oxidoreductase of *Yersinia pestis*. Using this experimentally determined structure as our template (Supplementary Figure S8A), we constructed a protein model of TVAG_091830 coding protein in atomic resolution. The structure geometry of the modeled protein was further validated by Ramachandran plot analysis, showing that 90.7% of residues were accommodated in the most favored regions (Supplementary Figure S9A). Additionally, the ERRAT score value (97.55%) exceeded the ideal score value (95%) (Supplementary Figure S9B), indicating that the predicted model is in high resolution. ProSA-web further evaluated the protein structure, and the resulting Z-score of −7.73 also confirmed the high quality of the modeled structure (Supplementary Figure S9C). We have successfully simulated nine different conformations of the MTZ ligands that fit into the active sites on the modeled protein with similar binding affinity around −5.0 kcal/mol (Supplementary Figure S8B and Supplementary Table S18), which indicate that MTZ is likely to compete with FAD for the same binding site (Supplementary Figure S8A). To conclude, MTZ might be considered as a potential inhibitor of MdaB, as it occupies the same binding pocket of FAD and eventually reduces the enzymatic activity of this protein.

## 4. Discussion

As the reliance on a single class of antimicrobial drugs for treating *T. vaginalis* infections may heighten vulnerability to the emergence of drug resistance, novel therapeutic agents are needed. However, the molecular mechanisms governing MTZ resistance of *T. vaginalis* are complicated, and previous studies mainly focused on the roles of a particular set of genes [14,18,38,39]. In this study, we sought to expand our knowledge of MTZ resistance in *T. vaginalis* and thus conducted large-scale RNA-seq analysis of multiple MTZ-R and MTZ-S strains in the presence or absence of MTZ, identifying strain-specific and common genes associated with MTZ resistance at the isoform level. We unraveled previously uncharacterized genes potentially correlated with MTZ resistance in MTZ-R isolates. Additionally, several genes and biological pathways were identified in MTZ-S *T. vaginalis* in response to MTZ-induced stress or cell death stimuli, paving the way for future investigations on the molecular mechanisms of cell death in trichomonads.

We reassessed the expression patterns of previously identified genes involved in MTZ resistance, such as PFOR, FR1, Fd, and iron-regulated genes, demonstrating the distinct expression levels of each isoform. Although earlier studies indicated that the mRNA levels of hydrogenosomal proteins PFOR, hydrogenase, Fd, and malic enzyme were reduced by up to 100% in MTZ-R isolates [38,40], our results showed inconsistent gene expression patterns of different PFOR and Fd isoforms in various MTZ-R strains. The findings are consistent with our previous proteomics study [22] and reports in MTZ-R *Entamoeba* [41,42] and *Giardia* [43], indicating that multiple molecules or alternative pathways modulate MTZ resistance in different drug-resistant isolates. Iron homeostasis has been shown to be related to MTZ resistance in *Helicobacter pylori* [44], *Bacteroides fragilis* [45], and *T. vaginalis* [18]. For instance, the ferrous iron transporter (feoAB) deficiency, which leads to reduced cellular iron transport, is linked with MTZ resistance in *B. fragilis*. We herein showed that several putative drug-resistant genes highly expressed under ID condition were also upregulated in the MTZ-R transcriptomes, such as MDR proteins, MDR pump, and Na^+^-driven efflux pump, suggesting that these genes may participate in regulating MTZ resistance of parasites upon iron deficiency. Hence, it is feasible to validate the roles of these ID-enriched genes in developing MTZ resistance of *T. vaginalis* under low iron environments by genetic manipulation, which will elucidate the MTZ resistance mechanism induced by low iron.

ABC transporters are indispensable components for the maintenance of cellular functions in all eukaryotes and prokaryotes, including parasites. It has recently drawn our attention that ABC transporters are involved in drug resistance in parasites and thus play essential roles in the control of parasitic diseases [46]. While the roles of ABC transporters and the related proteins in drug resistance have been investigated in several protozoans, including *Plasmodium* spp [47], *Toxoplasma gondii* [48], *Leishmania donovani* [49], *Trypanosoma brucei* [50], and *E. histolytica* [51], little is known about the role of ABC transporters in MTZ resistance of *T. vaginalis*. A previous study predicted that *T. vaginalis* possesses 98 putative ABC proteins, with the expansion of the ABCA and absence of the ABCG and ABCC subfamilies [52]. Additionally, some members of ABC transporters have been shown to be localized in the ER [52] and on the surface of *T. vaginalis* [53]. In the present study, we found that different isoforms of ABC transporters were distinctly expressed in various MTZ-R strains in the presence or absence of MTZ, implying their functional roles in the regulation of MTZ resistance. A transcriptomic study in *Giardia* consistently identified ABC transporters to be enriched among upregulated genes in one of the three MTZ-R lines 713-r [37]. Additionally, a metal ABC transporter (TVAG_254060) has also been shown to overexpress in another three MTZ-R *T. vaginalis* strains [21]. Since few members of ABC transporters were downregulated in all MTZ-R strains, we do not exclude the possibility that certain ABC transporters in *T. vaginalis* may have other cellular functions regulating a variety of transport processes, as illustrated in *L. tropica* [54]. For example, we showed that abcb9, an ABC subfamily B member 9 (ABCB9), was nearly not expressed in all MTZ-R strains but its expression is significantly induced in all MTZ-S strains upon MTZ treatment. It has been demonstrated that the expression of ABCB9 is lower in colorectal and ovarian tumors than normal tissues and is associated with poor survival of ovarian cancer [55,56]. Additionally, overexpression of human ABCB9 in yeast exhibits high sensitivity to valinomycin, suggesting that ABCB9 is important for conferring a high drug-sensitive phenotype [57]. Hence, functional verification of the ABC transporters derived from our transcriptomic data will shed light on the novel roles of this protein family in the MTZ resistance of *T. vaginalis*.

*T. vaginalis* G3 genome identified 911 BspA-like entries containing leucine-rich repeats (LRR) motif, representing the most enormous gene family encoding potential extracellular proteins for the pathogen [20]. BspA proteins have been shown to be universal to trichomonads, but specifically expanded in *T. vaginalis* [58]. Functional characterization of the BspA protein (TVAG_240680) revealed that it could remarkably increase the adherence of *T. vaginalis* to vaginal epithelial cells via a so far unknown mechanism [58]. We identified that several BspA-like genes were upregulated in the MTZ-R transcriptomes, suggesting their potential involvement in drug resistance. *E. histolytica* BspA (EhBspA1) has been shown to be the most upregulated gene in the trifluoromethionine-resistant strain, and overexpression of EhBspA1 indeed enhances the resistance to trifluoromethionine but does not increase the adhesive capacity of the transformant [59]. However, our results are inconsistent with previous work that showed downregulation of the 14 BspA-like genes in another three MTZ-R *T. vaginalis* strains [21]. It is possible that different MTZ-R strains express various isoforms of BspA-like proteins with multifaceted functions in *T. vaginalis*. Hence, the association between BspA-like proteins and MTZ resistance in *T. vaginalis* requires further investigations, and our datasets provide the candidate targets of such a large gene family for future functional verification.

Several shared upregulated or downregulated genes among all the MTZ-R transcriptomes were not previously investigated in *T. vaginalis*. For example, we found that 5′-nucleotidase surE is the most upregulated gene shared among all the MTZ-R transcriptomes; 5′/3′-nucleotidase surE has been shown to play a pivotal role in stress response and is required for the survival of *Escherichia coli* in the stationary growth phase [60]. As the function of 5′/3′-nucleotidase surE has not been characterized in parasites, it remained to be determined whether overexpression of 5′-nucleotidase surE in MTZ-R *T. vaginalis* maintains the survival of parasites in response to MTZ-induced stress. Additionally, different isoforms of Na^+^-driven multidrug efflux pump have been shown to be upregulated in the specific MTZ-R strain, and one of them is shared by all MTZ-R transcriptomes. Similarly, MDR pumps (TVAG_291970 and TVAG_210540) have also been demonstrated to be consistently upregulated in another three MTZ-R strains [21]. All these MDR-related proteins have multidrug and toxic compound extrusion (MATE)-like domain. MATE was initially identified as the factor contributing to MDR in *Vibrio parahaemolyticus* [61] and has attracted significant attention due to its role in the MDR of *Staphylococcus aureus* [62]. It has also been reported that a MATE-like sodium antiporter is uniquely upregulated in the MTZ-R *Giardia* 106-r line [37], and thus, the MATE transmembrane pump system is proposed to expel MTZ as sodium flaws back into the cell. Hence, it is worth investigating whether these MATE-containing proteins identified from this study may serve as the critical drug efflux pumps on the surface of *T. vaginalis* to drive MTZ resistance.

Three isoforms of putative MdaB are uniquely upregulated in the MTZ-S Tv-50148 strain, whereas only one isoform is upregulated in the MTZ-R Tv-50142 strain. It is noteworthy that these genes were markedly upregulated in the MTZ-S strains treated with MTZ, suggesting their vital roles responsible for drug-induced stress stimuli. Conserved domain analysis indicated that the genes encoding MdaB are putative NADPH-dependent FMN reductase, NAD(P)H-dependent oxidoreductase, or NADPH-quinone reductase with flavodoxin domains. Our results are consistent with the finding in *Giardia*, which indicates that FMN-dependent quinone reductases are downregulated in all analyzed *Giardia*-resistant lines [37]. It is worth mentioning that these oxidoreductase-related genes have similar functional domains to FR1, which has been proven to modulate MTZ resistance in *T. vaginalis* [16,17], suggesting that they may also serve as the critical oxygen scavenging enzymes to reduce the intracellular oxygen concentrations. Hence, we proposed that MTZ-S *T. vaginalis* strains expressed more genes encoding oxygen scavenging enzymes upon MTZ treatment to downregulate the intracellular oxygen levels, leading to parasites more susceptible to MTZ and probably causing cell death.

We reported for the first time the global gene expression changes and modulation of biological processes in MTZ-susceptible parasites encountering MTZ-induced stress and cell death stimuli. We found that the transcription of most genes involved in nucleotide metabolism (purine and pyrimidine metabolisms) and DNA replication are dramatically suppressed in MTZ-S parasites upon drug treatment (Supplementary Figures S2 and S3), possibly leading to cell death. This was supported by the previous work in *Clostridium bifermentans* [63] and *Bacteroides fragilis* [64], indicating that the primary action of MTZ is a rapid inhibition of DNA replication. We also observed an unexpected activation of the proteolytic pathways comprising ERAD, proteasome, and ubiquitin-mediated proteolysis in MTZ-susceptible parasites upon drug treatment. Accumulation of misfolded or unfolded proteins in the ER lumen, known as ER stress, might be harmful to cells. Hence, ER-specific stress response pathways such as unfolded protein response (UPR) and ERAD are activated to maintain proteostasis within the ER [65]. However, it should be noted that most of the UPR genes are missing in the *T. vaginalis* genome [66] (Supplementary Figure S5), suggesting that enhanced ERAD combined with the ubiquitin–proteasome system (UPS) for proteolysis is likely a vital process for parasites encountering MTZ-induced stress. It remains to be determined whether functional ERAD exists in *T. vaginalis*, as illustrated in trypanosomes [67], and how ERAD is linked to cell death in trichomonads lacking the apoptotic machinery.

## 5. Conclusions

Given that no alternative drugs are approved for the treatment of refractory trichomoniasis, we urgently need to better understand the MTZ resistance mechanisms and identify potential new drug targets for drug-resistant *T. vaginalis*. The present study utilizes deep RNA-seq to dissect the complex transcriptomes of MTZ-R strains compared with those of MTZ-S strains in the presence or absence of MTZ. To the best of our knowledge, this study represents the most detailed analysis of the transcriptional changes between MTZ-R and MTZ-S *T. vaginalis* strains. While the mechanisms of MTZ resistance have been widely discussed for more than 20 years, the major findings in *T. vaginalis* still focus on the well-known genes or pathways. We believe that multiple MTZ resistance mechanisms exist in different *T. vaginalis*-resistant strains, which can be supported by the comparison of different MTZ-R transcriptome datasets in our study and others [21]. Specifically, genes encoding different isoforms of ABC transporters and MDR-related proteins that are differentially expressed in the MTZ-R *T. vaginalis* strains have been highlighted in our study. Functional enrichment analysis revealed that nucleotide metabolisms are inhibited in parasites upon MTZ treatment, which is accompanied by activation of multiple cytoprotective responses, such as processes with regard to DNA repair and ERAD. These newly identified genes and biological pathways potentially associated with MTZ resistance and cell death deserve further investigations in the years ahead and will significantly contribute to a better understanding and ultimate prevention of MTZ resistance in eukaryotic pathogens.

## Figures and Tables

**Table 1:** Shared DEGs in all MTZ-R transcriptomes compared with those of MTZ-S.

Shared Upregulated Genes in the MTZ-R Transcriptomes			
Gene ID	MTZ-R/MTZ-S (log2FoldChange)	Padj	TrichDB Annotation
TVAG_195930	11.214	0	5′-nucleotidase surE
TVAG_154640	9.198	0	leucine-rich repeat protein, BspA family
TVAG_407240	9.146	0	60S ribosomal protein L10
TVAG_355510	8.626	0	leucine-rich repeat protein, BspA family
TVAG_219680	8.463	0	guanylate cyclase beta 1 subunit
TVAG_177180	7.166	0	WD-repeat protein
TVAG_195940	7.125	0	acyltransferase
TVAG_387850	6.553	0	ankyrin repeat-containing protein
TVAG_113870	5.905	0	acetyl-CoA hydrolase
TVAG_233960	5.346	0	vps9-ankyrin repeat-containing protein
TVAG_043070	5.156	0	fructose-bisphosphate aldolase
TVAG_333180	4.864	0	ankyrin repeat-containing protein
TVAG_011440	4.644	0	2-5A-dependent ribonuclease
TVAG_241690	4.58	0	ankyrin repeat domain protein
TVAG_156760	4.125	0	clan MA, family M8, leishmanolysin-like metallopeptidase
TVAG_410370	3.616	0	MYB81
TVAG_446260	3.599	0	guanylate cyclase
TVAG_277640	3.498	0	2-5A-dependent ribonuclease
TVAG_338210	3.305	0	leucine-rich repeat protein, BspA family
TVAG_005050	3.217	0	ankyrin repeat domain protein
TVAG_103510	3.207	0	leucine-rich repeat protein, BspA family
TVAG_316160	3.19	0	protein phosphatase-5
TVAG_108400	3.072	0.003	histidinol-phosphate aminotransferase
TVAG_114330	3.045	0	golgin IMH1
TVAG_396470	2.97	0.003	leucine-rich repeat protein, BspA family
TVAG_214470	2.868	0	leucine-rich repeat protein, BspA family
TVAG_005060	2.828	0	ankyrin repeat domain protein
TVAG_031910	2.788	0	calcium/calmodulin-dependent protein kinase
TVAG_271790	2.735	0	clan MA, family M8, leishmanolysin-like metallopeptidase
TVAG_315580	2.657	0.011	CAMK family protein kinase
TVAG_247080	2.572	0	adenylate cyclase
TVAG_157870	2.325	0	leucine-rich repeat protein, BspA family
TVAG_387720	2.281	0	ankyrin repeat-rich membrane-spanning protein
TVAG_262530	2.268	0	cortactin-binding protein
TVAG_161100	2.169	0	heat shock protein 70kD
TVAG_040090	2.163	0	cysteine synthase
TVAG_361490	2.143	0.025	CAMK family protein kinase
TVAG_397320	2.116	0	adenosine diphosphatase
TVAG_062550	2.106	0	clan SB, family S8, subtilisin-like serine peptidase
TVAG_426540	2.105	0	AGC family protein kinase
TVAG_185150	2.041	0	CMGC family protein kinase
TVAG_109730	2.04	0.012	40S ribosomal protein S23
TVAG_151620	2.008	0	heat shock protein 70 (HSP70)-4
TVAG_422640	2.006	0	26S proteasome non-ATPase regulatory subunit
TVAG_465230	1.974	0	dihydrodiol dehydrogenase
TVAG_099320	1.884	0.002	fibroblast growth factor, BASic, antisense
TVAG_339630	1.883	0	thioredoxin
TVAG_072440	1.882	0	coiled-coil domain-containing protein
TVAG_475160	1.827	0	HSP90 co-chaperone
TVAG_475150	1.821	0	AGC family protein kinase
TVAG_483040	1.784	0	Na^+^-driven multidrug efflux pump
TVAG_336370	1.76	0	pc-MYB2
TVAG_099020	1.754	0.156	eukaryotic translation initiation factor 4g
TVAG_383560	1.644	0	chaperone protein DNAj
TVAG_057020	1.615	0	60S ribosomal protein L22
TVAG_440390	1.604	0	CAMK family protein kinase
TVAG_284390	1.507	0	leucine-rich repeat protein, BspA family
TVAG_457350	1.498	0.016	r2r3-MYB transcription factor
TVAG_391100	1.439	0	clan MA, family M8, leishmanolysin-like metallopeptidase
TVAG_494730	1.347	0	lipid A export ATP-binding/permease protein msba
TVAG_359170	1.332	0	serine/threonine protein kinase
TVAG_201990	1.316	0	ubiquitin protein ligase E3a
TVAG_052020	1.289	0	PIKK family atypical protein kinase
TVAG_031010	1.289	0	triadin
TVAG_150550	1.236	0	DNAj/HSP40
TVAG_212040	1.137	0	sua5 protein
TVAG_143490	1.11	0	glycogen debranching enzyme
TVAG_368970	1.027	0	peptidylprolyl isomerase
**Shared Downregulated Genes in the MTZ-R Transcriptomes**			
TVAG_462960	−4.027	0	ankyrin
TVAG_602270	−3.429	0	aldo-keto reductase
TVAG_TEG_DS113597_1_3	−3.086	0	maverick transposable element conserved hypothetical protein
TVAG_357190	−2.748	0	calcium-dependent protein kinase
TVAG_524510	−2.735	0.005	helicase
TVAG_355170	−2.575	0	leucine-rich repeat protein, BspA family
TVAG_248020	−2.501	0	ankyrin repeat-containing protein
TVAG_197120	−2.461	0.002	ion channel nompc
TVAG_409820	−2.407	0	riboflavin kinase/fmn adenylyltransferase
TVAG_410410	−2.212	0	DNA double-strand break repair Rad50 ATPase
TVAG_473500	−2.175	0	inversin
TVAG_249030	−1.989	0	DNA double-strand break repair Rad50 ATPase
TVAG_002620	−1.73	0.097	ankyrin repeat-containing protein
TVAG_212110	−1.697	0	dynein light chain Tctex-type
TVAG_136630	−1.629	0	ankyrin repeat-containing protein
TVAG_455660	−1.564	0	clan CA, family C12, ubiquitin hydrolase-like cysteine peptidase
TVAG_290850	−1.451	0	casein kinase II beta chain
TVAG_095200	−1.25	0.001	RNA polymerase II ctd phosphatase

**Table 2:** Shared DEGs in all MTZ-R transcriptomes compared with those of MTZ-S in the presence of MTZ.

Shared Upregulated Genes in All MTZ-R Transcriptomes Treated with MTZ			
Gene ID	MTZ-R_MTZ/ MTZ-S_MTZ (log2FoldChange)	Padj	TrichDB Annotation
TVAG_177180	6.268	0	WD-repeat protein
TVAG_219680	5.775	0	guanylate cyclase beta 1 subunit
TVAG_195930	5.149	0	5′-nucleotidase surE
TVAG_019720	4.133	0	amino acid transporter
TVAG_396470	3.167	0	leucine-rich repeat protein, BspA family
TVAG_316160	3.112	0	protein phosphatase-5
TVAG_214840	2.522	0	CMGC family protein kinase
TVAG_038060	2.287	0	ubiquitin-conjugating enzyme E2-25kD
TVAG_254920	2.087	0.011	Na^+^-driven multidrug efflux pump
TVAG_397320	1.975	0	adenosine diphosphatase
TVAG_145340	1.823	0	alpha-galactosidase/alpha-N-acetylgalactosaminidase
TVAG_360900	1.499	0	bsu-protein phosphatase
**Shared Downregulated Genes in All MTZ-R Transcriptomes Treated with MTZ**			
TVAG_073330	−7.547	0	CAMK family protein kinase
TVAG_078520	−7.152	0	abcb9
TVAG_262830	−6.299	0	hydroxyacyl dehydrogenase
TVAG_262850	−5.986	0	short-chain dehydrogenases/reductase
TVAG_394080	−5.978	0	leucine-rich repeat protein, BspA family
TVAG_524510	−5.46	0	helicase
TVAG_273350	−4.943	0	r2r3-MYB transcription factor
TVAG_208780	−4.935	0	ankyrin repeat-containing protein
TVAG_RG_DS113355_8	−4.864	0	metallophosphoesterase domain-containing protein
TVAG_431960	−4.565	0	ABC transporter
TVAG_462960	−4.472	0.01	ankyrin
TVAG_309070	−3.952	0	ankyrin repeat-containing protein
TVAG_248020	−3.602	0	ankyrin repeat-containing protein
TVAG_048690	−3.558	0	aldo-keto reductase
TVAG_456680	−3.513	0	tankyrase
TVAG_060380	−3.503	0	5′->3′ exoribonuclease
TVAG_360070	−3.458	0	TKL family protein kinase
TVAG_477880	−3.449	0	CAMK family protein kinase
TVAG_343700	−3.408	0	ankyrin repeat-containing protein
TVAG_373800	−3.212	0	CAMK family protein kinase
TVAG_080130	−3.174	0	leucine-rich repeat protein, BspA family
TVAG_371570	−3.061	0	4-alpha-glucanotransferase
TVAG_248040	−2.897	0	actin
TVAG_262740	−2.857	0	ankyrin 2,3/unc44
TVAG_078510	−2.832	0	synaptonemal complex protein
TVAG_152410	−2.828	0	sugar transporter
TVAG_328000	−2.719	0.122	MYB-1
TVAG_078450	−2.615	0	leucine-rich repeat protein, BspA family
TVAG_329660	−2.585	0	alcohol dehydrogenase
TVAG_108070	−2.561	0	alpha-amylase
TVAG_318200	−2.544	0.001	r2r3-MYB transcription factor
TVAG_171590	−2.473	0.009	histone-lysine N-methyltransferase, bat/ehmt
TVAG_415420	−2.453	0	zinc-iron transporter
TVAG_473500	−2.365	0	inversin
TVAG_455660	−2.252	0	clan CA, family C12, ubiquitin hydrolase-like cysteine peptidase
TVAG_174020	−2.189	0.006	CAMK family protein kinase
TVAG_480660	−2.168	0	aromatic amino acid transporter
TVAG_050900	−2.131	0	myotrophin
TVAG_437170	−2.094	0	rolling pebbles
TVAG_425530	−2.091	0.019	CK1 family protein kinase
TVAG_457450	−2.087	0.001	KS1 protein precursor
TVAG_020440	−2.045	0	tankyrase
TVAG_096770	−2.032	0	ankyrin repeat-containing protein
TVAG_198600	−1.896	0	DNA polymerase alpha catalytic subunit
TVAG_479220	−1.8	0.003	heat shock protein
TVAG_198590	−1.698	0	homo sapiens cgi-128 protein
TVAG_254890	−1.441	0	pyruvate–flavodoxin oxidoreductase

## Data Availability

All data generated or analyzed in this study are included in this published article and its supplementary information files.

## Supplementary Materials

The following are available online at https://www.mdpi.com/article/10.3390/biomedicines9121817/s1. Supplementary Table S1: Identification of DEGs between the MTZ-R and MTZ-S transcriptomes. Supplementary Table S2: Identification of DEGs between the MTZ-R and MTZ-S transcriptomes upon treatment with MTZ. Supplementary Table S3: Identification of DEGs in the MTZ-S transcriptomes treated with MTZ compared with those in the untreated MTZ-S group. Supplementary Table S4: Differential expression of the previously identified hydrogenosomal genes involved in drug resistance in the MTZ-R transcriptomes compared with those of MTZ-S. Supplementary Table S5: Differential expression of the previously identified hydrogenosomal genes involved in drug resistance in the MTZ-R transcriptomes compared with those of MTZ-S in the presence of MTZ. Supplementary Table S6: Differential expression of iron-regulated genes between the MTZ-R and MTZ-S transcriptomes. Supplementary Table S7: Differential expression of iron-regulated genes between the MTZ-R and MTZ-S transcriptomes in the presence of MTZ. Supplementary Table S8: Shared DEGs in all MTZ-R transcriptomes compared with those of MTZ-S. Supplementary Table S9: Shared DEGs in all MTZ-R transcriptomes compared with those of MTZ-S in the presence of MTZ. Supplementary Table S10: Identification of uniquely upregulated genes in each MTZ-R strain. Supplementary Table S11: Identification of uniquely upregulated genes in each MTZ-R strain treated with MTZ. Supplementary Table S12: Identification of uniquely upregulated genes in each MTZ-S strain. Supplementary Table S13: Identification of uniquely upregulated genes in each MTZ-S strain treated with MTZ. Supplementary Table S14: Shared upregulated genes in all MTZ-S transcriptomes treated with MTZ compared with the untreated MTZ-S group. Supplementary Table S15: Shared downregulated genes in all MTZ-S transcriptomes treated with MTZ compared with the untreated MTZ-S group. Supplementary Table S16: Gene ontology enrichment analysis of the common DEGs in the MTZ-S transcriptomes treated with MTZ compared with the untreated MTZ-S group. Supplementary Table S17: KEGG pathway enrichment analysis of the common DEGs in the MTZ-S transcriptomes treated with MTZ compared with the untreated MTZ-S group. Supplementary Table S18: The list of predicted binding affinity of MTZ for the modeling structure of MdaB. Supplementary Figure S1: Differential expression of FR genes in the MTZ-R transcriptomes treated with or without MTZ. Supplementary Figure S2: Functional enrichment analysis identified the most downregulated pathways in MTZ-S parasites treated with MTZ. Supplementary Figure S3: Functional enrichment analysis identified the DNA replication pathway to be downregulated in MTZ-S parasites treated with MTZ. Supplementary Figure S4: Functional enrichment analysis identified the autophagy pathway to be downregulated in MTZ-S parasites treated with MTZ. Supplementary Figure S5: Functional enrichment analysis identified the protein processing in endoplasmic reticulum pathway to be upregulated in MTZ-S parasites treated with MTZ. Supplementary Figure S6: Functional enrichment analysis identified the mismatch repair pathway to be upregulated in MTZ-S parasites treated with MTZ. Figure S7. The most enriched pathways in MTZ-S parasites treated with MTZ compared with the untreated parasites. Figure S8. Proposed models for molecular docking of MTZ with MdaB. Supplementary Figure S9: Validation of the predicted model by different diagnostic methods.

## Abbreviations

MTZ	metronidazole
MTZ-S	MTZ-sensitive
MTZ-R	MTZ-resistant
ABC	ATP-binding cassette
MATE	multidrug and toxic compound extrusion
ERAD	endoplasmic reticulum-associated degradation
MdaB	modulator of drug activity B
HIV	human immunodeficiency virus
TNZ	tinidazole
PFOR	pyruvate: ferredoxin oxidoreductase
Fd	ferredoxin
ZIP	zinc regulated transporter, iron regulated transporter-like gene
Isc	Fe–S cluster
FeSOD	Fe-containing superoxide dismutase
SNPs	single nucleotide polymorphisms
SDW	sterile distilled water
RIN	RNA integrity number
NCBI	National Center for Biotechnology Information
PDB	Protein Data Bank
RMSD	root-mean-square deviation
PCA	principle component analysis
DEGs	differentially expressed genes
STK	succinate thiokinase
ID	iron-deficient
IR	iron-rich
MDR	multidrug resistance
GO	Gene Ontology
KEGG	Kyoto Encyclopedia of Genes and Genomes
feoAB	ferrous iron transporter
ABCB9	ABC subfamily B member 9
EhBspA1	*E. histolytica* BspA
UPR	unfolded protein response
UPS	ubiquitin–proteasome system
