# Supplementary material for: Dissecting the Transcriptomes of Multiple Metronidazole-Resistant and Sensitive Trichomonas vaginalis Strains Identified Distinct Genes and Pathways Associated with Drug Resistance and Cell Death
Source: Biomedicines. 2021 Dec 2;9(12):1817. doi: 10.3390/biomedicines9121817 (PMC8698965; doi:10.3390/biomedicines9121817)
Supplement: Supplementary file 1 [file biomedicines-09-01817-s001.zip › biomedicines-1449287-supplementary/Sup Figures_revised.pdf]

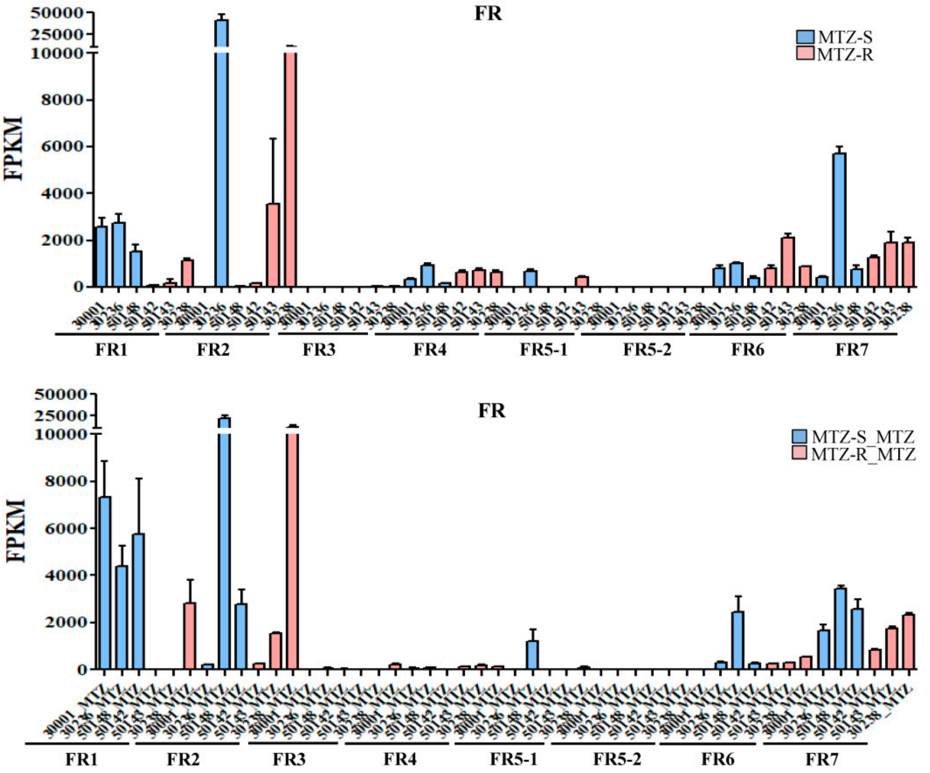

Figure S1. Differential expression of FR genes in the MTZ-R transcriptomes treated with or without MTZ. The expression levels of different FR isoforms (FR1-FR7) in the MTZ-S and MTZ-R strains treated with or without MTZ were analyzed and presented as mean ± SD of FPKM values obtained from three independent RNA-seq data.

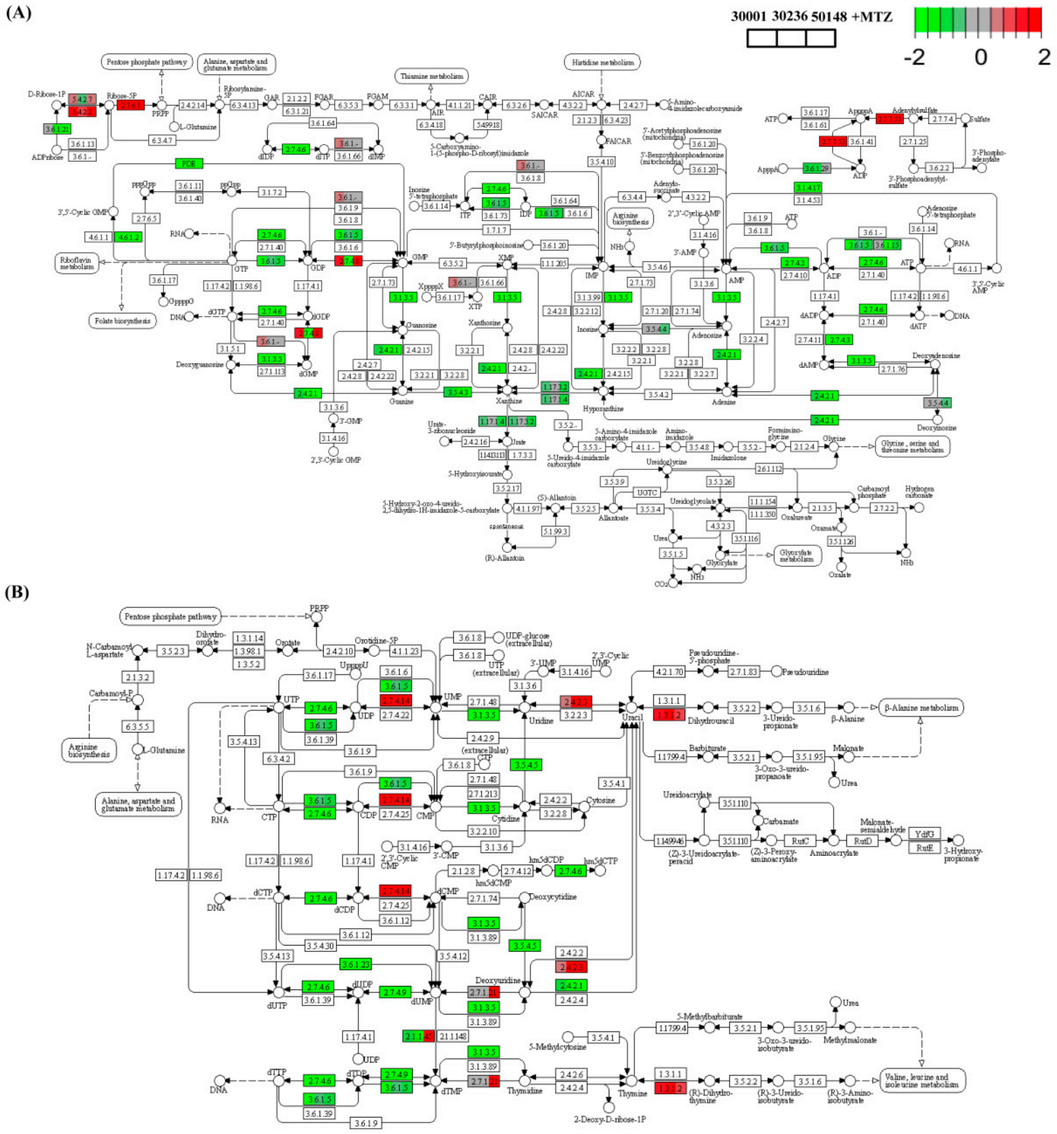

Figure S2. Functional enrichment analysis identified the most downregulated pathways in MTZ-S parasites treated with MTZ. KEGG enrichment analysis identified the downregulated pathways related to purine (**A**) and pyrimidine (**B**) metabolism in MTZ-S *T. vaginalis* transcriptomes treated with MTZ compared with those of the untreated MTZ-S isolates. The gene expression changes were presented as log2 fold change (FPKM of genes in each MTZ-S isolate (Tv-30001, Tv30236, and Tv-50148) treated with MTZ compared with that in the untreated isolate). The differential gene expression results from the three strains were combined into a particular gene. Genes shown in red and gene color indicate upregulation and downregulation, respectively.

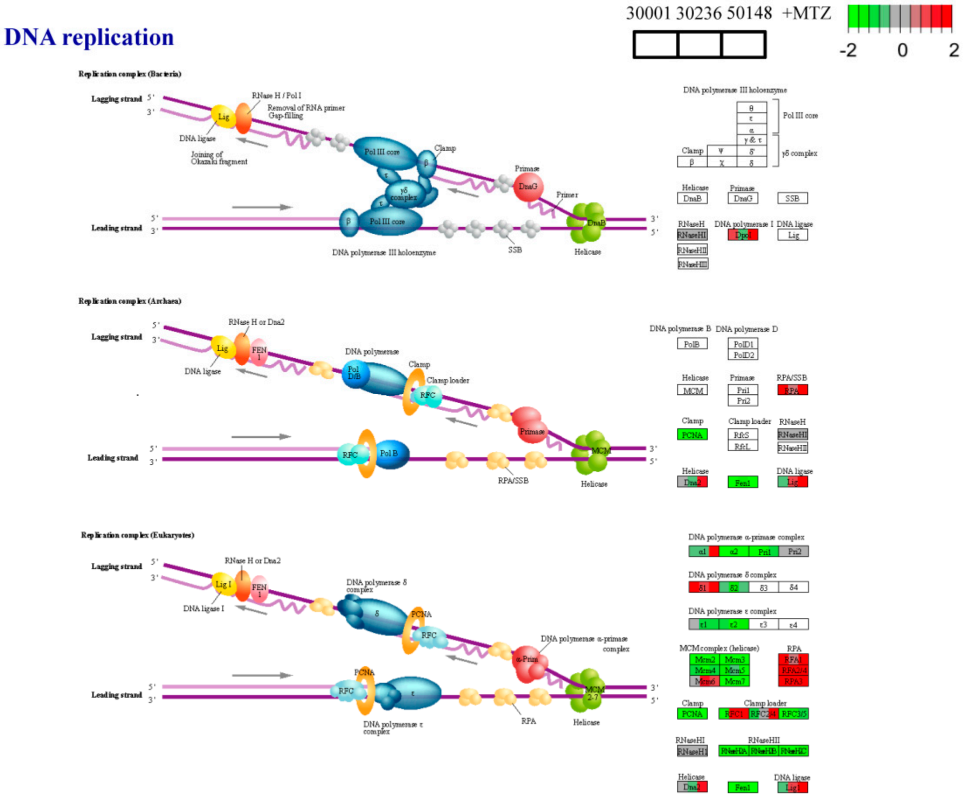

Figure S3. Functional enrichment analysis identified the DNA replication pathway to be down-regulated in MTZ-S parasites treated with MTZ. KEGG enrichment analysis identified the down-regulated pathway related to DNA replication in MTZ-S *T. vaginalis* transcriptomes treated with MTZ compared with that of the untreated MTZ-S isolates. The gene expression changes were presented as log2 fold change (FPKM of genes in each MTZ-S isolate (Tv-30001, Tv30236, and Tv-50148) treated with MTZ compared with that in the untreated isolate). The differential gene expression results from the three strains were combined into a particular gene. Genes shown in red and gene color indicate upregulation and downregulation, respectively.

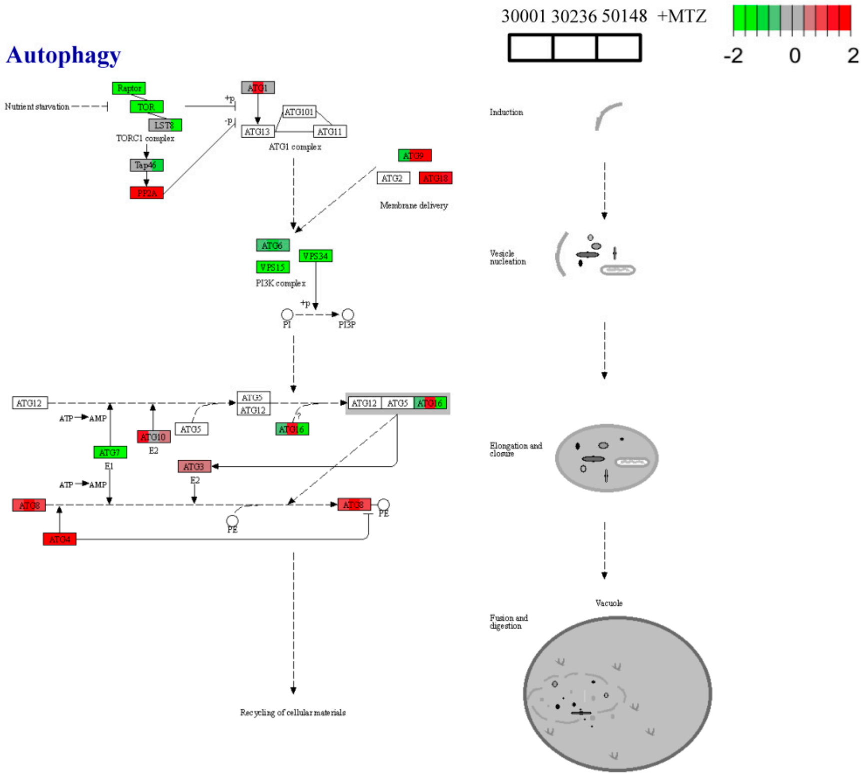

Figure S4. Functional enrichment analysis identified the autophagy pathway to be downregulated in MTZ-S parasites treated with MTZ. KEGG enrichment analysis identified the downregulated pathway related to autophagy in MTZ-S *T. vaginalis* transcriptomes treated with MTZ compared with that of the untreated MTZ-S isolates. The gene expression changes were presented as log2 fold change (FPKM of genes in each MTZ-S isolate (Tv-30001, Tv30236, and Tv-50148) treated with MTZ compared with that in the untreated isolate). The differential gene expression results from the three strains were combined into a particular gene. Genes shown in red and gene color indicate upregulation and downregulation, respectively.

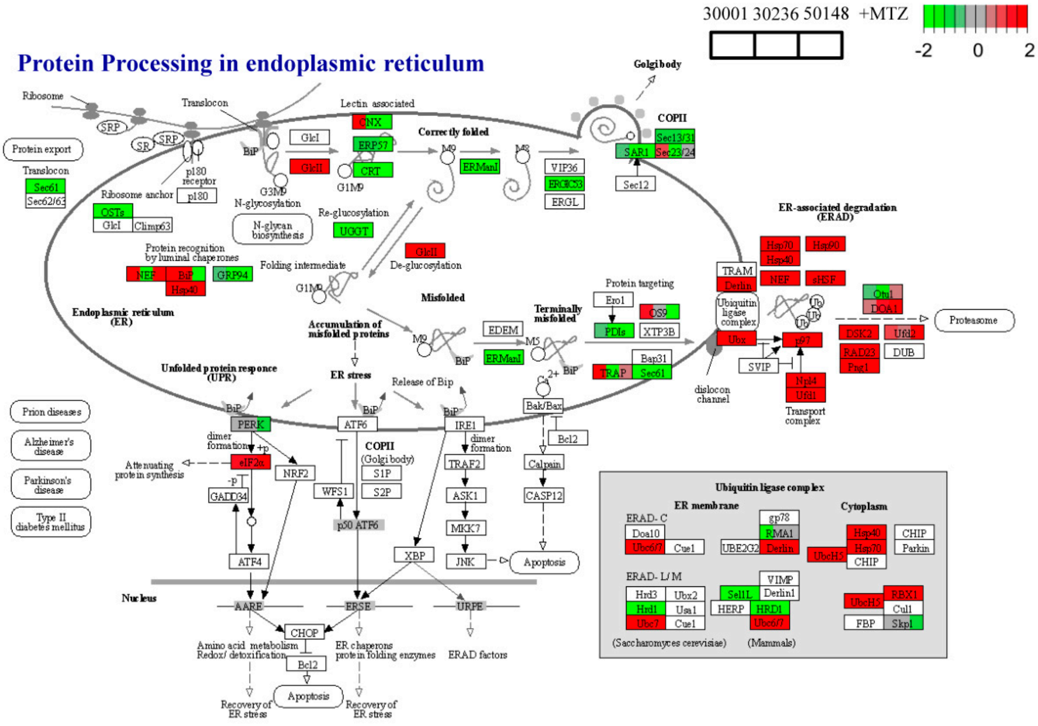

Figure S5. Functional enrichment analysis identified the protein processing in endoplasmic reticulum pathway to be upregulated in MTZ-S parasites treated with MTZ. KEGG enrichment analysis identified the downregulated pathway related to the protein processing in endoplasmic reticulum in MTZ-S *T. vaginalis* transcriptomes treated with MTZ compared with that of the untreated MTZ-S isolates. The gene expression changes were presented as log2 fold change (FPKM of genes in each MTZ-S isolate (Tv-30001, Tv30236, and Tv-50148) treated with MTZ compared with that in the untreated isolate). The differential gene expression results from the three strains were combined into a particular gene. Genes shown in red and gene color indicate upregulation and downregulation, respectively.

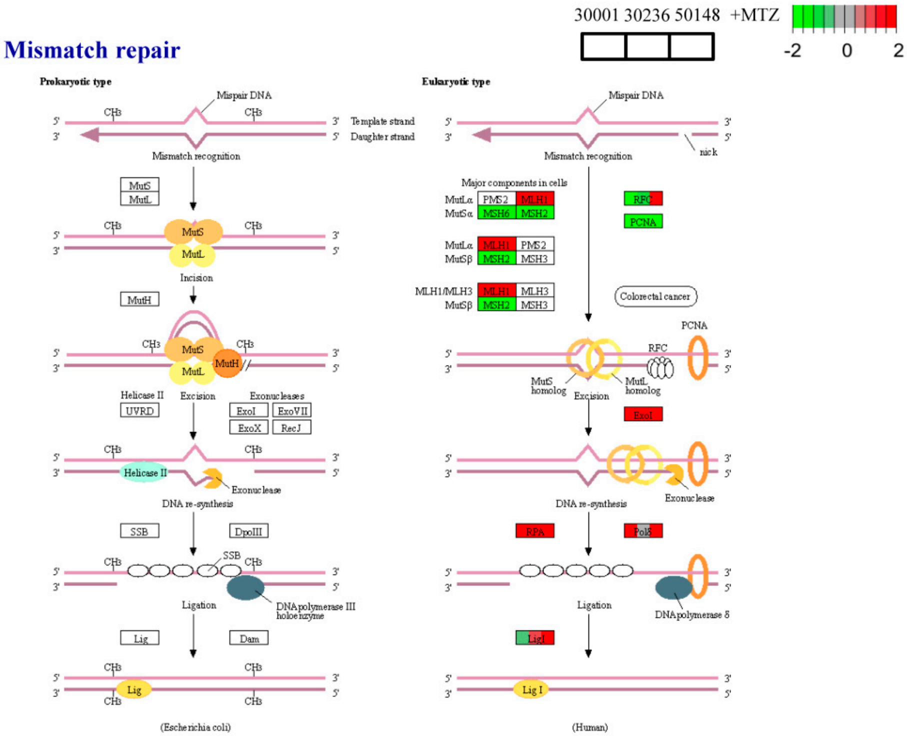

Figure S6. Functional enrichment analysis identified the mismatch repair pathway to be upregulated in MTZ-S parasites treated with MTZ. KEGG enrichment analysis identified the downregulated pathway related to the mismatch repair pathway in MTZ-S *T. vaginalis* transcriptomes treated with MTZ compared with that of the untreated MTZ-S isolates. The gene expression changes were presented as log2 fold change (FPKM of genes in each MTZ-S isolate (Tv-30001, Tv30236, and Tv-50148) treated with MTZ compared with that in the untreated isolate). The differential gene expression results from the three strains were combined into a particular gene. Genes shown in red and gene color indicate upregulation and downregulation, respectively.

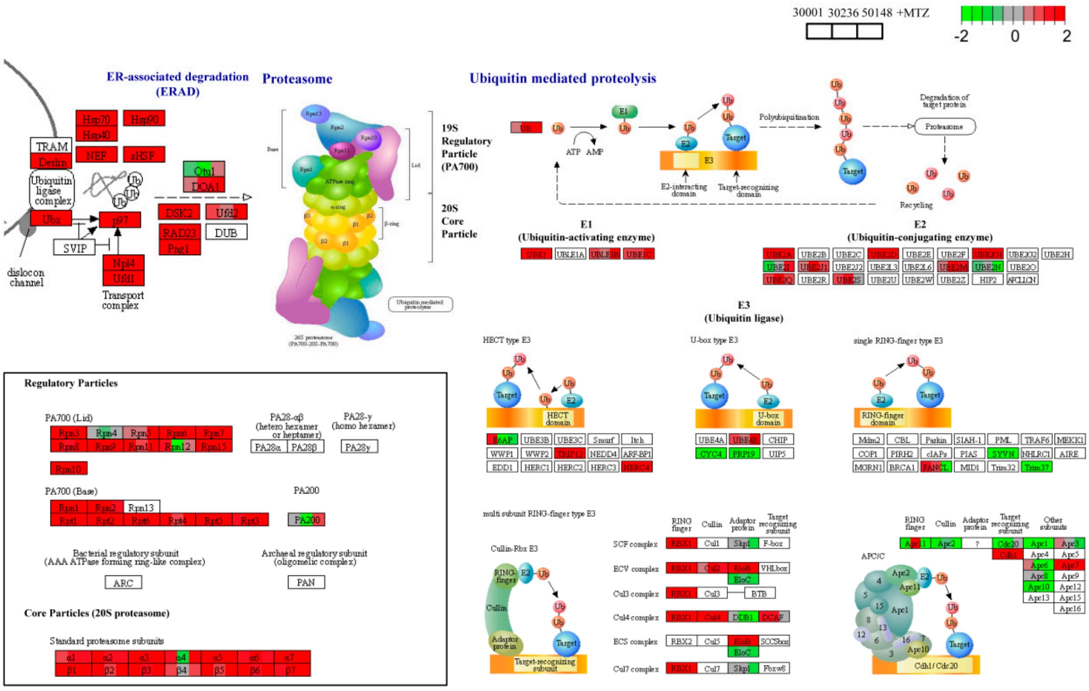

Figure S7. The most enriched pathways in MTZ-S parasites treated with MTZ compared with the untreated parasites. Functional enrichment analysis based on the KEGG database identified the most upregulated pathways related to proteolysis in MTZ-S T. vaginalis transcriptomes treated with MTZ compared with those of MTZ-S. ERAD is involved in the protein processing in the ER pathway. Proteasome contains regulatory particles and core particles. Ubiquitin-mediated proteolysis is comprised of E1 (ubiquitin-activating enzyme), E2 (ubiquitin-conjugating enzyme), and E3 (ubiquitin ligase). The gene expression changes were presented as log2 fold change (FPKM of genes in each MTZ-S isolate (Tv-30001, Tv30236, and Tv-50148) treated with MTZ compared with that in the untreated isolate). The differential gene expression results from the three strains were combined into a particular gene. Genes shown in red and gene color indicate upregulation and downregulation, respectively.

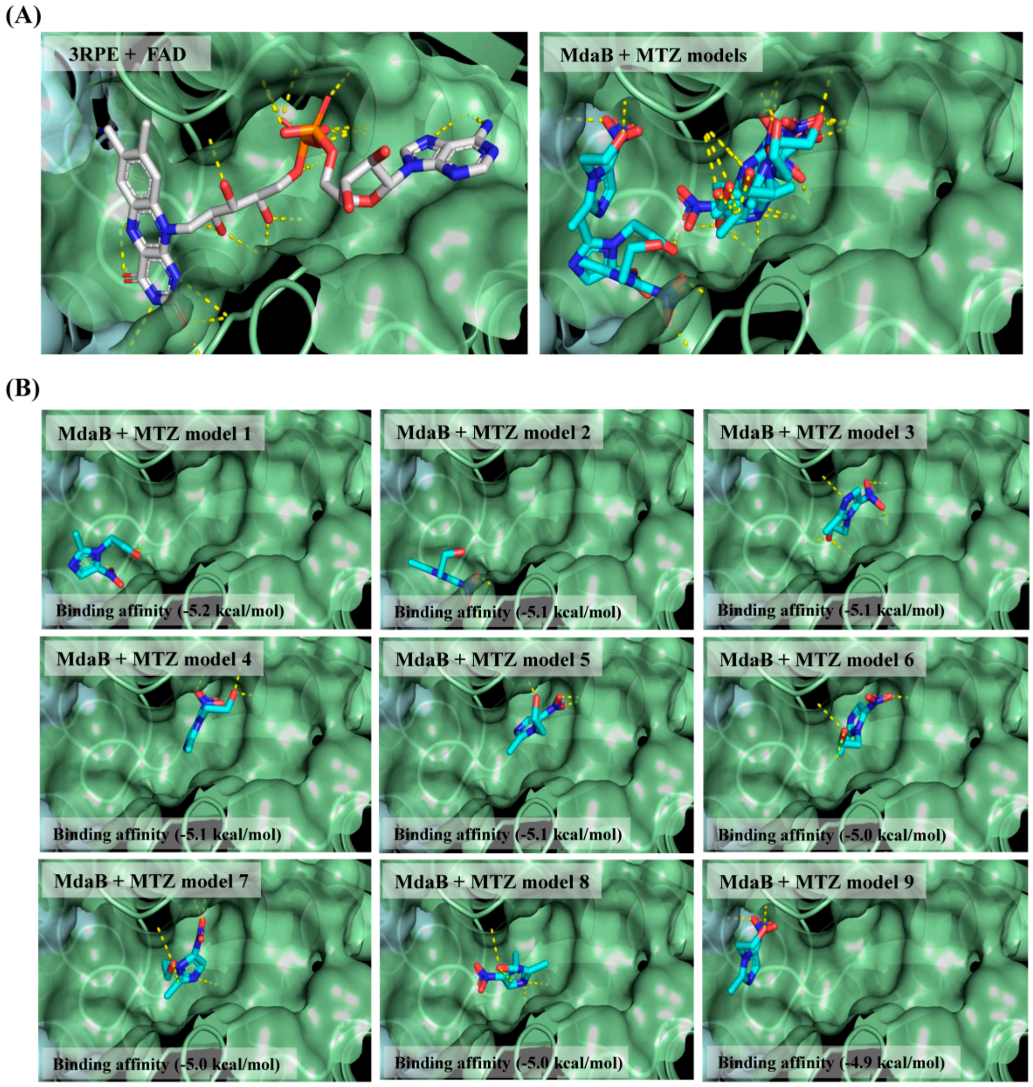

Figure S8. Proposed models for molecular docking of MTZ with MdaB. (**A**) MdaB was identified as a template structure based on high coverage and sequence similarity with the target protein, which is an experimentally determined FAD-MdaB crystal structure deposited in RCSB Protein Data Bank (PDB ID: 3RPE). Molecular docking analysis predicts all possible MTZ conformations that fit well into the same active site of the modeled structure established from the template structure of FAD-MdaB complex. (**B**) All the predicted models of the MTZ ligands represent sufficient binding strength with the target protein with binding affinity around -5.0 kcal/mol and fit well into the same binding site as FAD.

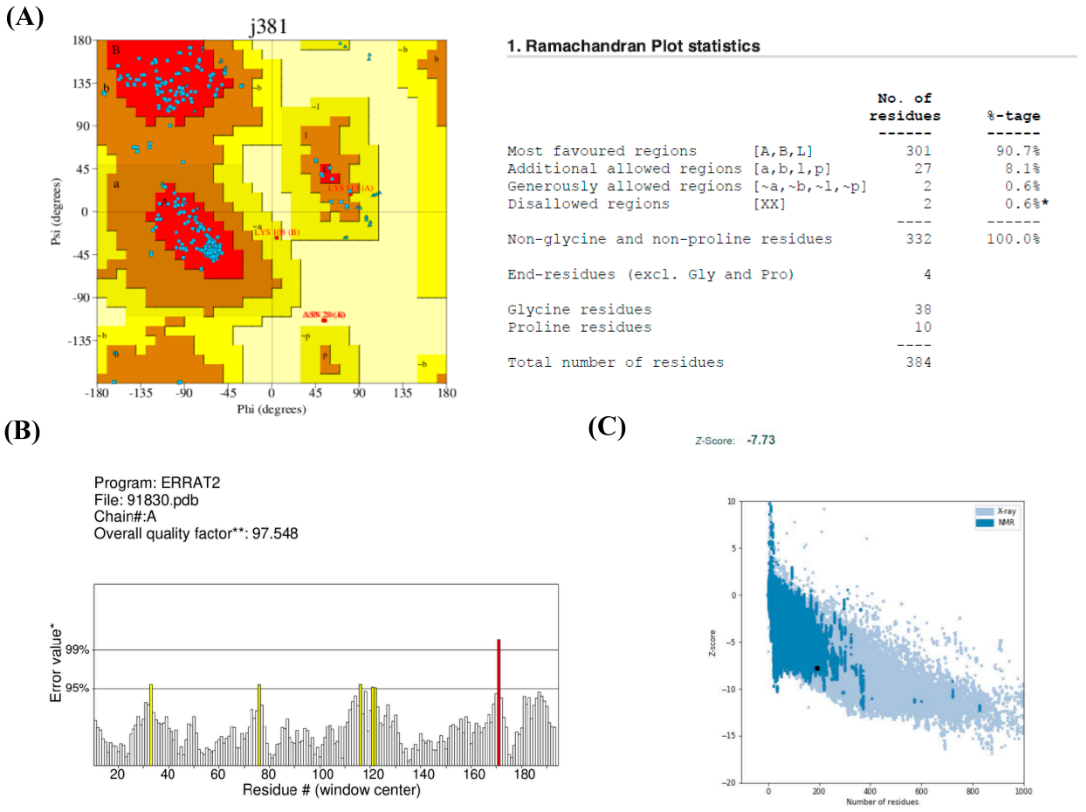

Figure S9. Validation of the predicted model by different diagnostic methods.(**A**) Ramachandran analysis shows that over 98.8% of the backbone dihedral angles from the modeled structure are located in energetically allowed regions. (**B**) The overall quality score of the modeled structure is 97.548, which is better than the ideal score value of 95%, based on analyzing the relative frequencies of noncovalent interactions between various atom types by ERRAT program. (**C**) ProSA is widely employed in the validation of protein structures obtained from experimentally determined methods and theoretical caculations. The Z-score indicates the overall quality of the modeled structure of MdaB and is highlighted as a black dot in the figure. As shown as a black dot in this figure, the overall model quality (Z-score) of the modeled structure of MdaB is within the range of scores characteristic for native proteins of similar size determined by X-ray crystallography (light blue) or NMR-spectroscopy (dark blue), deposited in RCSB Protein Data Bank.
